# Influence of Weather Variables and Plant Communities on Grasshopper Density in the Southern Pampas, Argentina

**DOI:** 10.1673/031.011.10901

**Published:** 2011-08-25

**Authors:** María Laura de Wysiecki, Marcelo Arturi, Sandra Torrusio, María Marta Cigliano

**Affiliations:** ^1^ Centro de Estudios Parasitológicos y de Vectores (CEPAVE), (CCT-La Plata- CONICET- UNLP), Calle 2 n° 584, 1900 La Plata, Argentina; ^2^ LISEA, Facultad de Ciencias Agrarias y Forestales, UNLP. Diagonal 113 n° 469, 1900 La Plata, Argentina; ^3^ Facultad de Ciencias Naturales y Museo, Avda 122 y 60. 1900 La Plata, Argentina.; ^4^ División Entomología, Museo de La Plata, Paseo del Bosque s/n. 1900 La Plata, Argentina

**Keywords:** Acrididae, community ecology, precipitation, vegetation, pampas

## Abstract

A study was conducted to evaluate the influence of weather (precipitation and temperature) and plant communities on grasshopper density over a 14-year period (1996–2009) in Benito Juárez County, Southern Pampas, Argentina. Total density strongly varied among plant communities. Highest values were registered in 2001 and 2003 in highly disturbed pastures and in 2002 and 2009 in halophilous grasslands. Native grasslands had the lowest density values. Seasonal precipitation and temperature had no significant effect on total grasshopper density. *Dichroplus elongatus* (Giglio-Tos) (Orthoptera: Acridoidea), *Covasacris pallidinota* (Bruner), *Dichroplus pratensis* Bruner, *Scotussa lemniscata* Stål, *Borellia bruneri* (Rehn) and *Dichroplus maculipennis* (Blanchard) comprised, on average, 64% of the grasshopper assemblages during low density years and 79% during high density years. *Dichroplus elongatus, S. lemniscata* and *C. pallidinota* were the most abundant species in 2001, 2002 and 2003, while *D. elongatus, B. brunneri* and *C.* *pallidinota* in 2009. *Dichroplus elongatus* and *D. pratensis*, mixed feeders species, were positively affected by summer rainfall. This suggests that the increase in summer precipitation had a positive effect on the quantity and quality forage production, affecting these grasshopper populations. *Scotussa lemniscata* and *C. pallidinota* were negatively affected by winter and fall temperature, possibly affecting the embryonic development before diapause and hatching. *Dichroplus elongatus* and *D. pratensis* were associated with highly disturbed pastures, *S. lemniscata* with pastures and *B. bruneri* and *D. maculipennis* with halophilous grasslands. *Covasacris pallidinota* was closely associated with halophilous grasslands and moderately disturbed pastures. Weather conditions changed over the years, with 2001, 2002 and 2003 having excessive rainfall while 2008 and 2009 were the driest years since the study started. We suggest that although seasonal precipitation and temperature had no significant effect on total grasshopper density, these weather variables and plant communities had differential influence on the dominant grasshopper species.

## Introduction

Grasshoppers are dominant native herbivores throughout the Pampas and, occasionally, they exhibit large temporal oscillations in abundance reaching high densities that cause extensive damage to grasslands and crops in the region (Cigliano et al. 2000; 2002; Carbonell et al. 2006).

Extensive research has been conducted on grasshopper dynamics to understand the underlying mechanism promoting the initiation of outbreaks and to assess strategies for long-term management of these insects (Pfadt 1977; Joern 1982; 2004; Joern and Pruess 1986; Joern and Gaines 1990; Kemp 1987, 1992; Kemp and Dennis 1993; Capinera and Thompson 1987; Johnson and Worobec 1988; Belovsky and Joern 1995; Cigliano et al. 1995, 2002; Onsager 2000; Gebeyehu and Samways 2003; Joern and Mole 2005; Branson et al. 2006). Several studies have found correlation between grasshopper abundance and weather variables, but different patterns have emerged from them in different regions of the world (Gage and Mukerji 1977; Capinera and Horton 1989; Fielding and Brusven 1990; Joern and Gaines 1990; Belovsky and Slade 1995; Powell et al. 2007; Branson 2008). Weather might also influence factors that may create density-dependence effects such as food availability and quality, vulnerability to predators and parasitoids and susceptibility to diseases (Capinera 1987; Joern and Gaines 1990). Ovadia and Schmitz (2004) found that although weather certainly affects demographic responses, negative feedbacks inherent in natural populations ultimately control the direct contribution of weather in determining population dynamics for most of the population cycle. Branson et al. (2006) indicated that climate clearly interacts with biotic factors, especially in its effect on food, plant availability and quality.

Previous studies in Benito Juárez county allowed the determination of a close association between the most abundant grasshopper species (*Dichroplus elongatus* (Giglio-Tos) (Orthoptera: Acridoidea), *Dichroplus pratensis* Bruner, *Scotussa lemniscata* Stål and *Covasacris pallidinota* (Bruner)), and plant communities with different degrees of disturbance history (Torrusio et al. 2002). The temporal changes in grasshopper communities based on the spatiotemporal characteristics of grasshopper density trends were recorded over a 5-year period (1997–2001). This was the first statistically demonstrated grasshopper outbreak, reported in Argentina (Cigliano et al. 2002).

In order to provide more information about new aspects of these insect pests for future management programs, the objective of this study was to determine whether the weather variables (precipitation and temperature) and plant communities influenced the variation in total and specific grasshopper densities, over a 14-year period, in the Southern Pampas.

## Materials and Methods

### Study area

**Figure 1.**
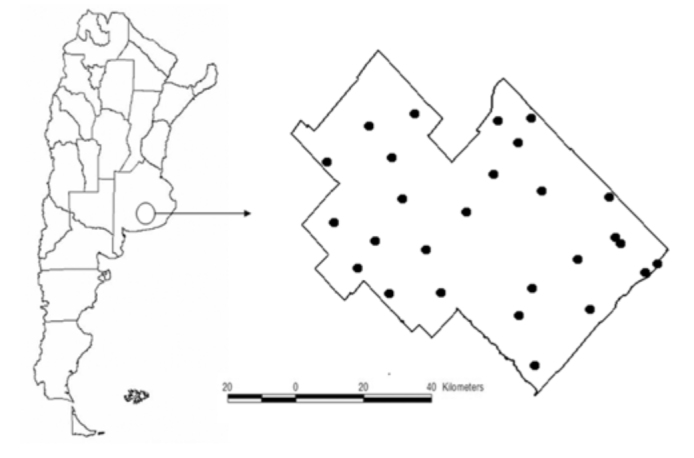
Location of study area: Benito Juárez County, Buenos Aires Province, Argentina. High quality figures are available online.

The study area was located in Benito Juárez county (530.772 ha), southeast of Buenos Aires province, from 60° 30′ W to 59° 15′ W and from 37° 15′ S to 38° 00′ S, in the Southern Pampas phytogeographic sub region (Cabrera and Willink 1973) (Figure 1). Mean temperatures are 21° C in summer and 7° C in winter and the average annual precipitation ranges from 700 mm in the west to 800 mm in the east. The area is flat and is principally used for crops production (winter and summer crops, covering 28 % of the area), and livestock production (pastures and rangelands covering 60%), with the exception of some hilly portions (10%), where pristine vegetation can still be found. The dominant native vegetation formerly consisted of perennial grasses (mainly species of *Stipa* and *Piptochaetium*) (Soriano 1992).

Twenty sites were selected in 1996, expanded to 27 in 1998 and held constant through 2009 (Figure 1). When any site was replaced by culture, it was not sampled. Sites were classified into five categories according to the dominant vegetation which reflected disturbance history (given in Torrusio et al. 2002): native grasslands are dominated by native grasses such as *Stipa caudata, Stipa neesiana, Stipa plumosa, Piptochaetium stipoides, Piptochaetium medium* and *Paspalum quadrifarium*; halophilous grasslands are compred of a short grass steppe dominated by a sparse cover of the grass *Distichlis spicata* (Soriano 1992); pastures have seeded grasses and dicots (annual and perennial, *Avena sp., Melilotus officinalis, Medicago sativa, Lolium multiflorum, Thynopyrum ponticum*. Moderately disturbed pastures and highly disturbed pastures have native grasslands grazed by livestock and invaded by introduced weeds (perennial and annual forbs).

### Grasshopper sampling

Grasshoppers were sampled at each site in summer (mid-January) to maximize chances of species detection with different phenological patterns. Grasshopper density was estimated by the rings method developed by Onsager and Henry (1977). Species composition and relative abundance were determined from 200 net sweeps per site in each sampling period. Each sweep traversed an arc of 180° through the vegetation with a net as described by Evans (1984, 1988). Grasshoppers collected via sweep net were placed in plastic bags, kept in portable coolers, and taken to the laboratory for identification to species.

Sweep-net collections were used to estimate relative abundance of grasshopper species that was calculated as the abundance of species *i* relative to the total abundance of all species collected at each site × 100. The density of each species was calculated by multiplying the proportion of each species by overall grasshopper density. For the analysis, the six more abundant species (*D. elongatus*, *D. pratensis, Dichroplus maculipennis* (Blanchard), *S.* *lemniscata*, *Borellia bruneri* (Rehn) and *C. pallidinota*) were considered.

### Weather parameters

Seasonal (fall, winter, spring and summer) precipitation was used for the analysis and considering that most grasshopper species are univoltines, each season was related to the different life cycle stages of grasshoppers. Fall (April–June) and winter (July–September) seasons correspond to the eggs development, spring (October–December) to hatching and nymphal development and summer (January–March) to mating and oviposition. Seasonal average temperature data was also used for the analysis.

Spatio-temporal variation of total grasshopper density and grasshopper species density were evaluated using linear mixed models (Pinheiro and Bates 2000) in which sampling sites were used as random effects. Logarithmic transformed density was used as dependent variable. Mean temperature and seasonal precipitation at the sampling summer as well as at the previous summer, fall, winter and spring were included as independent variables. Temporarily and spatially lagged densities were included as autoregressive terms to account for temporal and spatial dependence (Haining 1990). A 1-year lagged density was used as temporal autoregressive term. Spatially lagged density was obtained as the product of the inverse distance matrix between all sampling sites by the vector density (Haining 1990). In this inverse weighting matrix the diagonal elements were set to zero, thus, this term represented the effect of grasshopper density in the surrounding sampling sites on the grasshopper density of a given sampling site. The same procedure was followed using the density of other species (total density - analyzed species density) in surrounding sampling sites on the density of each species analyzed. Thus, the effect of grasshopper density in the previous season and the effect of grasshopper density in the surrounding sampling sites were assessed. Plant communities were also included as predictors using dummy variables. A backward stepwise selection procedure was used to fit a fixed model with weather and spatially and temporally lagged densities. After that, sampling sites were taken as random effect in order to evaluate whether the intercept and slopes in the model were site specific or not. This analysis was carried out using the “nlme” package in R (Pinheiro and Bates 2000).

**Table 1.**
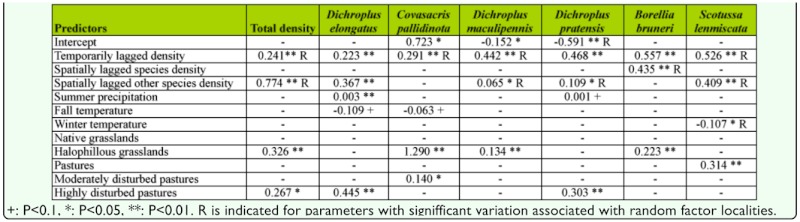
Models of total and specific density variations. Parameter estimates from the mixed models are indicated as well as the R-squared for the linear models attained with only the fixed components.

## Results

**Figure 2.**
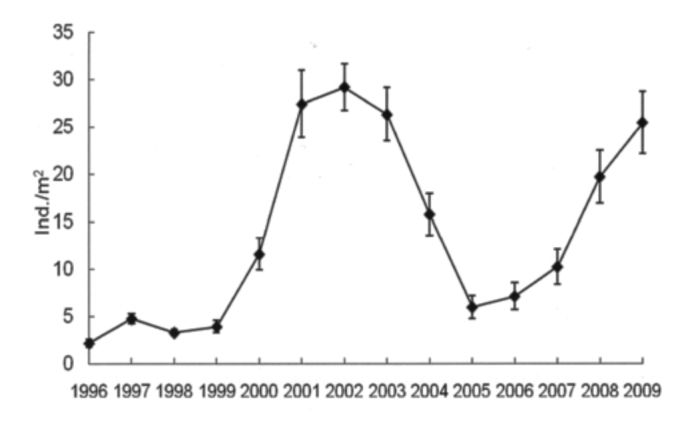
Total grasshopper density (ind/m^2^ ± SE), during the study (1996–2009), in Benito Juárez County, Southern Pampas, Argentina. High quality figures are available online.

Total grasshopper density varied during the study period. Higher densities were reached in 2001 (27.4 ind/m^2^), 2002 (29.2 ind/m^2^), 2003 (26.3 ind/m^2^) and 2009 (25.5 ind/m^2^), and lower densities in 1996–1999 and 2005–2007, with an average grasshopper density of 3.5 ind/m^2^ and 7.8 ind/m^2^, respectively (Figure 2).

**Figure 3.**
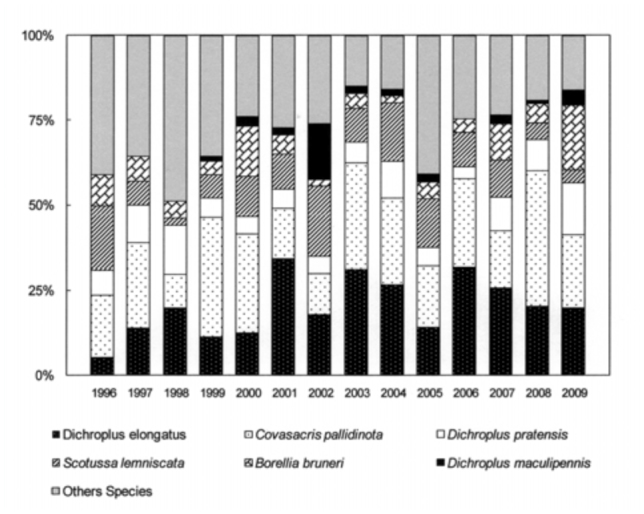
Relative abundance grasshopper, expressed in percentage, during the study (1996–2009), in Benito Juárez County, Southern Pampas, Argentina. High quality figures are available online.

The six most abundant species (*D. elongatus, C. pallidinota, D. pratensis, S. lemniscata, B. bruneri* and *D. maculipennis*) comprised, on average, 64% of the grasshopper assemblage during low density years and 79% during the high density years. *Dichroplus elongatus, S. lemniscata, C. pallidinota* and *D. maculipennis* were the most abundant species in 2001, 2002 and 2003, while *D. elongatus, B. brunneri* and *C. pallidinota,* were the most abundant in 2009 (Figure 3).

### Spatio-temporal variation

The data exhibited temporal and spatial dependence since a positive effect on grasshopper density in the previous summer, as well as the grasshopper density in the surrounding sampling sites were observed in all the analyses, except for *C. pallidinota* in which spatial dependence was not observed (Table 1).

Spatial dependence in species specific models exhibited effects of conspecific or non-conspecific grasshopper density, depending on the species analyzed. Models including sampling sites with specific intercepts were better fitted than those with a single intercept. Specific sampling sites slopes were required for different variables in all models except for *C. pallidinota.*

**Figure 4.**
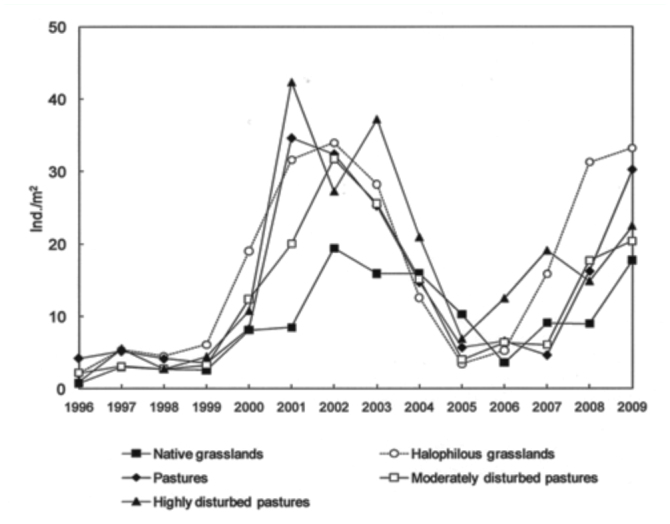
Total grasshopper density (ind/m^2^) in different plant communities, during the study (1996–2009), in Benito Juárez County, Southern Pampas, Argentina. High quality figures are available online.

Weather variables had different effects in the models for total and specific grasshopper densities, while differences among plant communities were observed in all cases. Seasonal precipitation and temperature had no significant effect on total grasshopper density. Total grasshopper density strongly varied among plant communities (Table 1). Highest values were registered in 2001 and 2003 in highly disturbed pasture and, in 2002 and 2009, in halophilous grasslands. Native grasslands had the lowest density values in all situations (Figure 4).

**Figure 5.**
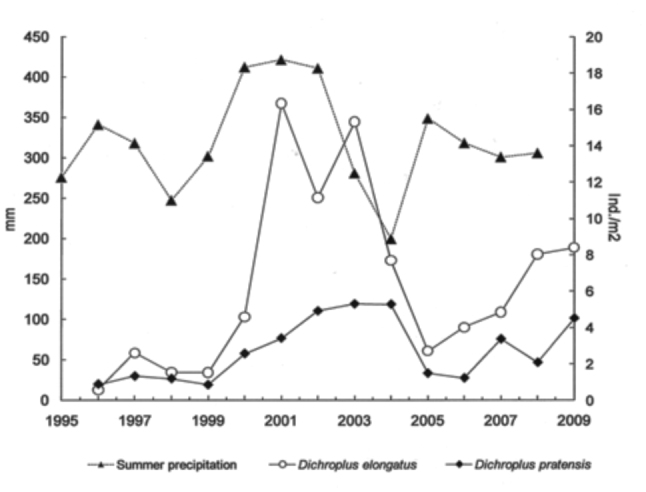
*Dichroplus elongatus* and *Dichroplus pratensis* densities (ind./m^2^) and summer precipitations (mm) during an 14 years period study (1996–2009), in Benito Juárez county, Southern Pampas, Argentina. High quality figures are available online.

**Figure 6.**
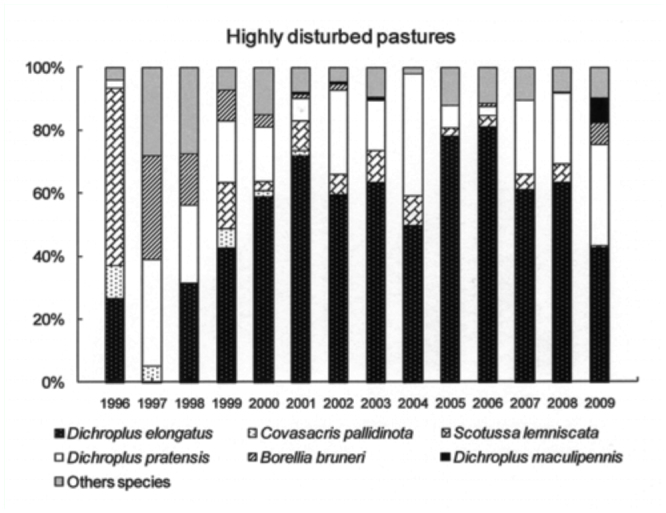
Relative abundance, expressed in percentage, of the six most abundant grasshopper species in Highly disturbed pastures, *Dichroplus elongatus, Dichroplus pratensis, Covasacris pallidinota, Scotusa lemniscata, Borellia bruneri* and *Dichroplus maculipennis*, during the study (1996–2009). High quality figures are available online.

**Figure 7.**
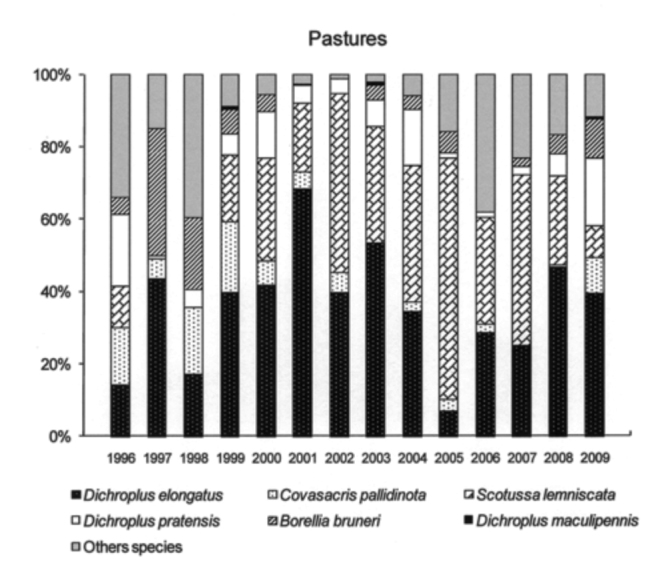
Relative abundance, expressed in percentage, of the six most abundant grasshopper species in Pastures, *Dichroplus elongatus, Dichroplus pratensis, Covasacris pallidinota, Scotusa lemniscata, Borellia bruneri* and *Dichroplus maculipennis*, during the study (1996–2009). High quality figures are available online.

**Figure 8.**
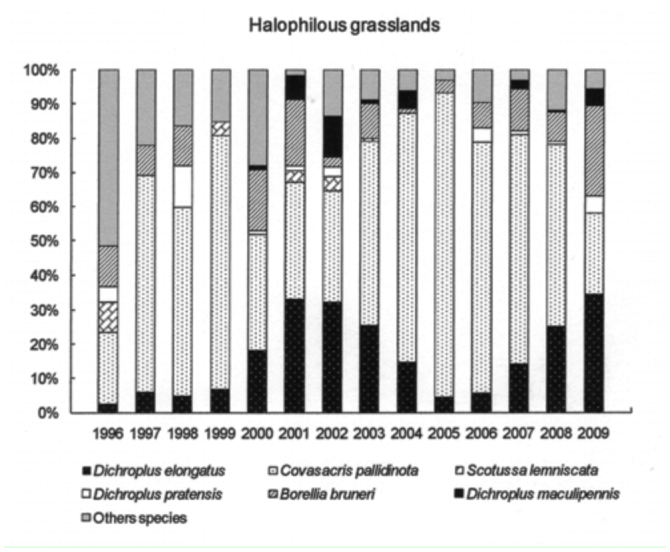
Relative abundance, expressed in percentage, of the six most abundant grasshopper species in Halophilous grasslands, *Dichroplus elongatus, Dichroplus pratensis, Covasacris pallidinota, Scotusa lemniscata, Borellia bruneri* and *Dichroplus maculipennis*, during the study (1996–2009). High quality figures are available online.

**Figure 9.**
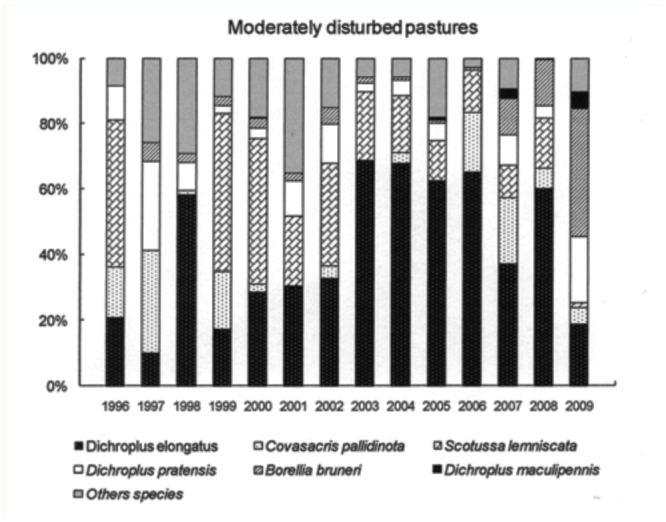
Relative abundance, expressed in percentage, of the six most abundant grasshopper species in Moderately disturbed pastures, *Dichroplus elongatus, Dichroplus pratensis, Covasacris pallidinota, Scotusa lemniscata, Borellia bruneri* and *Dichroplus maculipennis*, during the study (1996–2009). High quality figures are available online.

**Figure 10.**
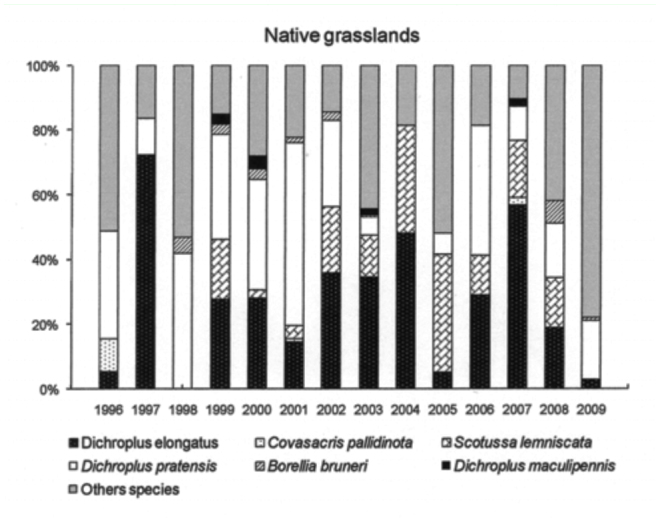
Relative abundance, expressed in percentage, of the six most abundant grasshopper species in Native grasslands, *Dichroplus elongatus, Dichroplus pratensis, Covasacris pallidinota, Scotusa lemniscata, Borellia bruneri* and *Dichroplus maculipennis*, during the study (1996–2009), in Benito Juárez County, Southern Pampas, Argentina. High quality figures are available online.

*Dichroplus elongatus* and *D. pratensis* were positively affected by summer precipitation (*p* <0.01 and *p* < 0.1, respectively) and strongly associated with highly disturbed pastures (*p* < 0.01) (Table 1, Figures 5 and 6). However only *D. elongatus* was slightly negatively affected by fall temperature (*p* < 0.1) (Table 1). *Scotussa lemniscata* was negatively affected by winter temperature (*p* < 0.05), and was strongly associated with pastures (*p* < 0.01) (Table 1, Figure 7). *Covasacris pallidinota* had a slightly negative relationship with fall temperature (*p* < 0.1), and was strongly associated with halophilous grasslands (*p* < 0.01) and moderately disturbed pastures (*p* < 0.05) (Table 1, Figures 8 and 9). *Borellia brunneri* and *D. maculipennis* were associated with halophilous grasslands (*p* < 0.05) (Table 1, Figure 8). There was no association between grasshopper species and native grasslands (Table 1, Figure 10).

## Discussion

In this study, all the models exhibited a strong positive effect of the density in the previous summer as well as of density in the surrounding sampling sites. Thus, weather variables explained the variation not associated to the spatio-temporal dependence of the population density. The best fit of models, including random intercepts or slopes, indicate the existence of sampling sites specific response to spatial and temporal lagged grasshopper density and weather variables. Grasshopper total density was not affected by seasonal precipitation and temperature. This result does not agree with those registered in Saskatchewan and Alberta, Canada, where grasshopper populations were negatively correlated with spring and summer precipitation (Gage and Mukerji 1977; Johnson and Worobec 1988; Powell et al. 2007). In the USA, different patterns emerged between weather conditions and grasshopper densities: in the South, densities tended to decrease in hot and dry conditions, while in the North they tended to increase under such situations (Capinera 1987; Capinera and Thompson 1987; Capinera and Horton 1989; Belovsky and Joern 1995; Fielding and Brusven 1990).

Grasshopper total density was positively associated with highly disturbed pastures and halophilous grasslands. Halophilous grasslands consist of a short grass steppe dominated by a sparse cover of the perennial salty-lowland grass *D. spicata*, growing in poorly drained sodic soils with nutrient deficiencies (Hurtado et al. 2005). In the same area Torrusio et al. (2002) recorded that highly disturbed pastures had more plant species than halophilous grasslands, with an average of 8.2 and 4.2 species, respectively. The plants registered in highly disturbed pastures included exotic perennial forbs (*Carduus acanthoides, Taraxacum officinale, Centaurea solsistialis, Amni majus*, etc), and some seeded species and native grasses *(Piptochaetium medium, Stipa formicarum, S. trichotoma, Bromus brevis*), while in halophilous grasslands, *D. spicata* was the dominant grass followed by other halophilous species like *Hordeum euclaston, Puccinellia glaucescens, Sisyrinchium sp* and *Spergula vilosa.* Both communities had bare ground (5% and 15% in highly disturbed pastures and halophilous grasslands, respectively). The largest number of plants of highly disturbed pastures may promote the presence of mixed-feeders species while the halophilous grasslands may favor the presence of grass-feeders species. In addition, bare ground enables the warming of the sites and this situation may have a positive impact in different aspects of the grasshopper life (egg hatching, growth and development rates).

*Dichroplus elongatus* and *D. pratensis* densities were positively affected by summer precipitations. Precipitation is the major factor controlling above ground primary production in temperate grasslands. Above ground net primary production is strongly influenced by the amount and distribution of annual precipitation (Lauenroth 1979; Sala et al. 1988, Sala 2001). Also, nitrogen use efficiency increases with increasing precipitation (Burke el al. 1997). It was expected that an increase in summer precipitation had a positive effect on the forage availability and quality in the studied sites. Belovsky and Joern (1995) indicated that plant availability and quality is one of the mechanisms that changes over time and between sites, and this process impacts on density-independent survival and reproduction as well as determining the strength for food competition. Joern and Behmer (1997), using defined diets under controlled laboratory conditions for the grass-feeding grasshopper *Ageneotettix deorum,* and found that nitrogen in significant concentrations impacted adult weight gain, egg production rate, the elapsed time until the first egg pod and the time between the first and the second egg pod. The same authors (1998) evaluated the survival and reproduction of adult females of *Melanoplus sanguinipes* (mixed-feeder) and *Phoetaliotes nebrascensis* (grass-feeder) in response to defined diets that varied in total nitrogen and total carbohydrate and they found that both species responded quite differently and concluded that, although host plant quality can contribute significantly to grasshopper population response, a uniform explanation is not possible.

*Scotussa lemniscata* and *C. pallidinota* were slightly negatively affected by winter and fall temperatures. Several studies have demonstrated that soil temperature affects embryonic development before the diapause and determines the end of diapause, thereby influencing egg hatching time (Capinera and Sechrist 1982; Kemp and Sanchez 1987; Fisher et al. 1996).

When plant communities were considered, *D. elongatus* and *D. pratensis* were shown to be associated with highly disturbed pastures and *S.* *lemniscata* with pastures (seeded grasses), while *B. bruneri* and *D. maculipennis* were associated with halophilous grasslands and *C. pallidinota* was closely associated with halophilous grasslands and moderately disturbed pastures.

*Dichroplus elongatus* and *D. pratensis* are historically two of the most harmful grasshopper species in Argentina (Carbonell et al. 2006). They are mixed-feeders species, mostly fed on grass and dicots (Gangwere and Ronderos 1975; de Wysiecki and Sánchez 1992).

*Scotussa lemniscata* is typically associated with moist environments with dense and relatively high vegetation, like pastures (seeded grasses) (Torrusio et al. 2002, Carbonell et al. 2006). Contrary to most other grasshoppers, it lays the egg-pods on the stems of grasses (Cigliano and Ronderos 1994).

*Covasacris pallidinota* and *B. brunneri* are oligophagous and grass-feeder species, the former, almost exclusively fed on *D. spicata* while the latter feeds on a few grasses (*H. euclaston, D. spicata, Agropyron elongatun,* and *Stipa formicarun*) (Mariottini 2009). *Borellia bruneri* is a common grassland species, which thrives in areas of sparse vegetation with patches of bare soil, mostly found in rather dry localities with a good cover of short grasses and less abundant where the vegetation is dense and tall (Carbonell 1995).

*Dichroplus maculipennis* is considered historically one of the most damaging grasshoppers in Argentina, especially in areas of the Pampas and Patagonia. It is a polyphagous species and prefers to lay the egg-pods on low and poorly drained soils, with low and sparse vegetation (Lange et al. 2005; Carbonell et al. 2006), such as halophilous grasslands. Several studies conducted during the 90s' in the Pampas suggested an apparent decrease in the abundance of its populations (Cigliano et al. 1995), and it was usually recorded as a rare species (Cigliano et al. 2000; De Wysiecki et al. 2004), but in December, 2008 and January, 2009, an outbreak of *D. maculipennis* of historical magnitude (densities up to 75 ind/m^2^ and development of adult mass displacements), occurred in 20 counties in the central, southern and southwest pampas, affecting more than 2,500,0000 ha (unpublished observations). These years were the driest of the last 47 years and the losses produced by this grasshopper were in the millions (Mariottini 2009). Benito Juárez was one of these counties, but at that time, this grasshopper only affected 26,800 ha that was concentrated in two areas not sampled in this study, mainly dominated by halophilous grasslands (unpublished observations).

All during the study, 2001, 2002, 2003 and 2009 were outbreak years. In 2001 and 2003 the highest densities were found in highly disturbed pastures and *D. elongatus, S. lemniscata* and *C. pallidinota* represented 59.5% and 62.5% of the grasshoppers collected, respectively. In 2002, and 2009 the highest densities were recorded in halophilous grasslands, and *D. elongatus, S. lemniscata* and *D. maculipennis* represented 55% of the grasshoppers collected in the former year and *D. elongatus, B. bruneri* and *C. pallidinota* 60% in the latter.

Climatic conditions changed during the study, with excessive rainfall in the years 2001, 2002 and 2003 while 2008 and 2009 were the driest years since the study started. Considering these events we can suggest that the weather conditions have had an influence on some plant communities and on the dominant species. In 2002 *S. lemniscata* represented about 20.5% but only 3.9 % in 2009, while *B. bruneri* in 2002 represented 2% and 19.1 % in 2009. Mariottini (2009), reported the decrease in *S. lemniscata* densities during 2009 in Laprida county, in the west boundary of Benito Juárez, and this was related to the unusual dry and hot conditions of season 2008–2009 and the concomitant almost complete lack of green coverage that might also partly explain the decrease in this species as a lack of the appropriate vegetation would mean the absence of needed substrates for egg-pod laying sites. Also, the extremely dry conditions may have negatively affected the embryonic development of the eggs.

As regards *B. bruneri*, the sparse vegetation and the bare ground could promote soil warming and affect some demographic aspects of this grasshopper that impact positively its density.

Grasshopper species do not react equally and may show marked differences in temporal responses to various factors. In spite of the fact that weather variables and plant communities have clear influence on the life cycle of these insects, they cannot be considered independently of biotic factors, such as predation, parasitism, and pathogen incidence, on which they also depend.

Ritchie (2000) found that mixed-feeder grasshopper density was positively correlated with plant tissue N and with soil N, but negatively related to previous year's precipitation. For grass-feeders, density was negatively correlated with soil N and not significantly related to precipitation. Although in this study there were no field experiments to evaluate the relationships raised by Ritchie (2000), *D. elongatus* and *D. pratensis* (mixed-feeder grasshoppers) could respond favorably to forage quality and quantity increase due to summer rainfall. With respect to *B. bruneri* (grass-feeder grasshopper), one of the most abundant species in 2009, that was the driest year, the warm temperature due to the sparse vegetation and the bare ground may have affected some demographic aspect affecting its density.

In this study where two grasshopper outbreaks were recorded, one in 2001 reported by Cigliano et al. (2002) that continued for two more years (2002–2003) and the other in 2009, we found that seasonal precipitation and temperature had no significant effect on total grasshopper density. However, these weather variables plus plant communities had differential influence on the dominant grasshopper species.

## References

[bibr01] Belovsky GE, Joern A, Cappuccino N, Price P (1995). The dominance of different regulating factors for rangeland Grasshoppers.. *Population Dynamics: New Approaches and Synthesis*.

[bibr02] Belovsky GE, Slade GE (1995). Dynamics of two Montana grasshopper populations: relationships among weather, food abundance and intraspecific competition.. *Oecologia*.

[bibr03] Branson DH (2008). Influence of a large late summer precipitation event on food limitation and grasshopper population dynamics in a Northern Great Plains grassland.. *Environmental Entomology*.

[bibr04] Branson DH, Joern A, Sword GA (2006). Sustainable management of insect herbivores in grassland ecosystems: new perspectives in grasshopper control.. *BioScience*.

[bibr05] Burke IC, Lauenroth WK, Parton WJ (1997). Regional and temporal variation in net primary production and nitrogen mineralization in grasslands.. *Ecology*.

[bibr06] Cabrera A, Willink A (1973). Biogeografía de América Latina. Monografía 13. Serie de Biología. Secretaria General de Organización de los Estados Americanos..

[bibr07] Capinera JL, Capinera JL (1987). Population ecology of rangeland grasshoppers.. *Integrated Pest Management on Rangeland: A shortgrass Prairie perspective*.

[bibr08] Capinera JL, Sechrist TS (1982). Grasshoppers (Acrididae) of Colorado: Identification, Biology and Management.. *Colorado Agricultural Experiment Station Bulletin* 584S..

[bibr09] Capinera JL, Thompson D (1987). Dynamics and structure of grasshopper assemblages in shortgrass prairie.. *Canadian Entomologist*.

[bibr10] Capinera JL, Horton DR (1989). Geographic variation in effects of weather on grasshopper infestation.. *Environmental Entomology*.

[bibr11] Carbonell C (1995). Revision of the tribe Scyllinini, nov. (Acrididae: Gomphocerinae), with descriptions of new genera and species.. *Transactions of the American Entomological Society*.

[bibr12] Carbonell C, Cigliano MM, Lange CE (2006). *Especies de acridomorfos (Orthoptera) de Argentina y Uruguay.*.

[bibr13] Cigliano MM, Ronderos RA (1994). Revision of the South American Grasshopper Genera *Leiotettix* Bruner and *Scotusa* Giglio-Tos (Orthoptera, Acrididae, Melanoplinae).. *Transactions of the American Entomologial Society*.

[bibr14] Cigliano MM, Kemp WP, Kalaris TM (1995). Spatiotemporal analysis of regional outbreak in rangeland grasshoppers (Orthoptera:Acrididae).. *Journal of Orthoptera Research*.

[bibr15] Cigliano MM, de Wysiecki ML, Lange CE (2000). Grasshopper (Orthoptera: Acrididae) species diversity in the pampas, Argentina.. *Diversity and Distributions*.

[bibr16] Cigliano MM, Torrusio SE, de Wysiecki ML (2002). Grasshopper (Orthoptera: Acrididoidea) community composition and temporal variation in The Pampas, Argentina.. *Journal of Orthoptera Research*.

[bibr17] De Wysiecki ML, Sánchez N (1992). Dieta y Remoción de forraje de *Dichroplus pratensis* (Orthoptera, Acrididae) en un pastizal de la provincia de La Pampa, Argentina.. *Ecología Austral* 2.

[bibr18] De Wysiecki ML, Torrusio SE, Cigliano MM (2004). Caracterización de las comunidades de acridios del partido de Benito Juárez, sudeste de la provincia de Buenos Aires, Argentina.. *Revista de la Sociedad Entomológica Argentina*.

[bibr19] Evans EW (1984). Fire as a natural disturbance to grasshopper assemblages of tallgrass prairie.. *Oikos*.

[bibr20] Evans EW (1988). Grasshopper (Insecta: Orthoptera: Acrididae) assemblages of tallgrass prairie: influences of fire frequency, topography, and vegetation.. *Canadian Journal of Zoology*.

[bibr21] Fielding DJ, Brusven MA (1990). Historical analysis of grasshopper (Orthoptera: Acrididae) population responses to climate in Southern Idaho, 1950–1980.. *Environmental Entomology*.

[bibr22] Fisher JR, Kemp WP, Pierson FB, Wight JR (1996). *Grasshopper Egg Development: the Role of temperature in predicting Egg Hatch.* Cap. IV.2.. Grasshoppers Integrated Pest Manager User Handbook United States Departament of Agrigulture, Animal, and Plant Health Inspection Service, Technical Bulletin N° 1809..

[bibr23] Gage SH, Mukerji MK (1977). A perspective of grasshopper population distribution in Saskatchewan and interrelationship with weather.. *Environmental Entomology*.

[bibr24] Gandwere SK, Ronderos RA (1975). A synopsis of food selection in argentine Acridoidea.. *Acrida*.

[bibr25] Gebeyehu S, Samways MJ (2003). Responses of grasshopper assemblages to long-term grazing management in a semi-arid African Savanna.. *Agriculture, Ecosystems and Environment*.

[bibr26] Haining R. (1990). *Spatial data analysis in the social and environmental sciences.*.

[bibr27] Hurtado MA, Moscatelli GN, Godagnone RE, De Barrio R, Etcheverry RO, Caballé MF, Llambías E (2005). Los suelos de la provincia de Buenos Aires.. *Relatorio del XVI Congreso Geológico Argentino. Geología y Recursos Minerales de la provincia de Buenos Aires*.

[bibr28] Joern A (1982). Distributions, densities, and relative abundances of Grasshoppers (Orthoptera: Acrididae) in a Nebraska Sandhills prairie.. *The Prairie Naturalist*.

[bibr29] Joern A (2004). Variation in grasshopper (Acrididae) densities in response to fire frequency and bison grazing in tallgrass prairie.. *Environmental Entomology*.

[bibr30] Joern A, Pruess KP (1986). Temporal constancy in grasshopper assemblies (Orthoptera: Acrididae).. *Ecological Entomology*.

[bibr31] Joern A, Gaines SB, Chapman RF, Joern A (1990). Population dynamics and regulation in grasshoppers.. *Biology of grasshoppers*.

[bibr32] Joern A, Behmer S (1997). Importance of dietary nitrogen and carbohydrates to survival, growth, and reproduction in adults of the grasshopper *Ageneotettix deorum* (Orthoptera: Acrididae).. *Oecologia*.

[bibr33] Joern A, Behmer S (1998). Impact of diet quality on demographic attributes in adult grasshoppers and the nitrogen limitation hypothesis.. *Ecological Entomology*.

[bibr34] Joern A, Mole S (2005). The plant stress hypothesis and variable responses by blue grama grass (*Bouteloua gracilis*) to water, mineral nitrogen, and insect herbivory.. *Journal of Chemical Ecology* vol.

[bibr35] Johnson DL, Worobec A (1988). Spatial and temporal computer analysis of insects and weather: grasshoppers and rainfall in Alberta.. *Memoirs of the Entomological Society of Canada*.

[bibr36] Kemp WP (1987). Probability of outbreak for rangeland grasshopper (Orthoptera: Acrididae) in Montana: application of Markovian principles.. *Economic Entomology*.

[bibr37] Kemp WP (1992). Temporal variation in rangeland grasshopper (Orthoptera: Acrididae) communities in the steppe region of Montana, USA.. *Canadian Entomologist*.

[bibr38] Kemp W.P., Sánchez N.E. (1987). Differences in post-diapause thermal requirements for eggs of two rangeland grasshoppers.. *Canadian Entomology*.

[bibr39] Kemp WP, Dennis B (1993). Density dependence in rangeland grasshoppers (Orthoptera: Acrididae).. *Oecologia*.

[bibr40] Lange CE, Cigliano MM, De Wysiecki ML, Barrientos Lozano L, Almaguer P (2005). Los acridoideos de importancia económica en la Argentina.. *Manejo integrado de la langosta centroamericana y acridoideos plaga en América Latina*.

[bibr41] Lauenroth WK, French NR (1979). Grassland primary production: North American grasslands in perspective.. *Perspectives in Grassland Ecology*.

[bibr42] Mariottini Y. (2009). Biología y ecología de acridios (Orthoptera: Acridoidea) del Sur de la región Pampeana. Tesis doctoral. Facultad de Ciencias Naturales y Museo..

[bibr43] Onsager JA (2000). Suppression of grasshoppers in the Great Plains through grazing management.. *Journal of Range Management*.

[bibr44] Onsager JA, Henry JE (1977). A method for estimating the density of rangeland grasshoppers (Orthoptera, Acrididae) in experimental plots.. *Acrida*.

[bibr45] Ovadia O, Schmitz OJ (2004). Weather variation and thopic interaction strengh: sorting the signal from the noise.. *Oecologia*.

[bibr46] Pinheiro JC, Bates DM (2000). *Mixed-Effects Models in S and S-Plus.*.

[bibr47] Powell LR, Berg AA, Johnson DL, Warland JS (2007). Relationships of pest grasshopper populations in Alberta, Canada to soil moisture and climatic variables.. *Agricultural and Forest Meteorology*.

[bibr48] Pfadt RE, Kulman HM, Chiang HC (1977). Some aspects of the ecology of grasshopper populations inhabiting the Shortgrass Plains.. *Insect Ecology papers presented in the A.C. Hodson Lectures.* U. Mn. Agr. Expt. Sta. Tech. Bul. 310..

[bibr49] Ritchie ME (2000). Nitrogen limitation and trophic vs abiotic influences on insect herbivores in a temperate grassland.. *Ecology*.

[bibr50] Sala OE, Roy J, Saugier B, Mooney HA (2001). Productivity of temperate grasslands.. Terrestrial Global Productivity.

[bibr51] Sala OE, Parton WJ, Lauenroth WK, Joyce LA (1988). Primary production on the central grassland region of the Unites States.. *Ecology*.

[bibr52] Soriano A., Coupland RT (1992). Río de La Plata Grasslands.. *Natural grasslands. Introduction and Western hemisphere. Ecosystems of the World*.

[bibr53] Torrusio S, Cigliano MM, de Wysiecki ML (2002). Grasshopper (Orthoptera: Acridoidea) and plant community relationships in the Argentine Pampas.. *Journal of Biogeography*.

